# Temperature, relative humidity and sunshine may be the effective predictors for occurrence of malaria in Guangzhou, southern China, 2006–2012

**DOI:** 10.1186/1756-3305-6-155

**Published:** 2013-05-30

**Authors:** Tiegang Li, Zhicong Yang, Ming Wang

**Affiliations:** 1 Guangzhou Center for Disease Control and Prevention, Guangzhou, Guangdong Province, 510440, China

**Keywords:** Malaria, Parasite disease, Meteorological variables, Correlation, Early warning

## Abstract

Malaria has been endemic in Guangzhou for more than 50 years. The goal of this study was to use a negative binomial regression to identify the relationship between meteorological variables and malaria reported. Our results revealed that each 1°C rise of temperature corresponds to an increase of 0.90% in the monthly number of malaria cases. Likewise, a one percent rise in relative humidity led to an increase of 3.99% and a one hour rise in sunshine led to an increase of 0.68% in the monthly number of cases. Our findings may be useful for developing a simple, precise malaria early warning system.

## Letter to the editor

Malaria is a protozoan disease caused by parasites of the genus *Plasmodium*. It is one of the leading causes of illness and death in the world [1], the vast majority of cases are in Africa and South-East Asia [2]. As the largest trading city in southern China, Guangzhou has over 7.94 million registered inhabitants and a 4.76 million floating population (from 2010 census data) [3], here malaria has been endemic for more than half a century [4], and public health authorities are concerned about its high prevalence.

In 2009, China launched a Malaria Elimination Action Plan (MEAP) for 2010–2020 [5]. Despite various prevention and intervention strategies that are implemented and enhanced by the government, the incidence of malaria still showed an increasing trend in Guangzhou. In 2012, a total of 112 malaria confirmed cases were reported, which is more than twice the number reported in 2009 (53 cases). Malaria control is a big challenge due to many factors. One of the critical programs to prevent this disease concentrated on monitoring and predicting malaria incidence. Previous studies have shown that weather factors had significant correlation with malaria [6-8]. However, due to the difference in geographical and climate characteristics, meteorological variables influence malaria incidence in different areas in different ways [9]. Moreover, in most recent years, reports regarding meteorological variables as predictors for the occurrence of malaria in southern China are fairly limited. In this study, we aim to examine the effect of weather variability on the incidence of malaria in the subtropical city of Guangzhou for the period of 2006–2012, and assist public health prevention and control measures.

## Methods

In China, malaria is a notifiable Class-B communicable disease, and all cases of malaria were diagnosed according to the unified diagnostic criteria issued by The Chinese Ministry of Health. Physicians who diagnose suspected or confirmed malaria cases must report these cases to Guangzhou Centers for Disease Control and Prevention (GZCDC) through the National Notifiable Disease Report System (NNDRS). We obtained the laboratory confirmed cases during the period of 2006 to 2012 from NNDRS, and simultaneous meteorological data, including daily average temperature (in degrees Centigrade), relative humidity (as a percentage), atmospheric pressure (in hPa), and wind velocity (in meters per second) were obtained from the documentation of the Guangzhou Meteorological Bureau(GZMB). A negative binomial regression was used to identify the relationship between meteorological variables and malaria. The cases are the prevalence of malaria per 100 000 inhabitants grouped by month of onset and for each meteorological variable, a monthly average or aggregate was calculated.

A preliminary analysis was conducted through Pearson’s correlation coefficient (‘r’) matrix within meteorological variables. This indicated that the model constructed using contemporaneously both temperature and atmospheric pressure suffered from collinearity problems, because the two variables showed strong negative correlation (r = −0.86, P < 0.01) (Table 1). Thus, we first carried out two separate negative binomial regression models: the first included average temperatures but no atmospheric pressure, while the second considered atmospheric pressure but no temperature data. In addition both models included relative humidity, wind velocity, sunshine, rainfall, and year as independent variables. The final model included only those variables that reached a p value of <0.10 in the preliminary model with all the variables. To quantify the effects of meteorological variables, we computed the influences (e^β^ − 1), which virtually correspond to the relative risks. These analyses were carried out using SAS (V.8.01, SAS Institute, Cary, New Jersey, USA). Data are presented as relative risk and corresponding 95% CI. P values <0.05 were considered statistically significant.

**Table 1:** Pearson’s correlation coefficient (‘r’) matrix of meteorological variables in Guangzhou, southern China, 2006-2012

	**Atmospheric pressure**	**Relative humidity**	**Average temperature**	**Rainfall**	**Sunshine**	**Wind velocity**
Atmospheric pressure	1					
Relative humidity	−0.59(p = 0.00)	1.00				
Average temp.	−0.86(p = 0.00)	0.32(p = 0.00)	1.00			
Rainfall	−0.58(p = 0.00)	0.52(p = 0.00)	0.53(p = 0.00)	1.00		
Sunshine	−.028(p = 0.01)	−0.43(p = 0.00)	0.40(p = 0.00)	−0.16(p = 0.15)	1.00	
Wind velocity	−0.10(p = 0.36)	0.20(p = 0.06)	−0.28(p = 0.01)	−0.12(p = 0.29)	0.00(p = 0.99)	1.00

## Results

From January 1, 2006 to December 31, 2012, a total of 560 malaria confirmed cases were reported in Guangzhou, southern China, of which 83.39% (467) were male patients and 16.61% (93) were female patients. The age ranged from 1 to 83 years old (mean age was 37.64). The proportion of confirmed cases between <5, 6–19, 20–44, 45–64, and >65 was 0.36%, 3.57%, 71.07%, 21.96%, and 3.047%, respectively.

Of those six meteorological variables studied, temperature, relative humidity and sunshine were statistically significant in the final model. Each 1°C rise of temperature corresponds to an increase of 0.90% (95% CI 0.60% to 1.10%) in the monthly number of malaria cases. Likewise, a one percent rise of relative humidity led to an increase of 3.99% (95% CI 2.53% to 5.48%) and a one hour rise in monthly duration of sunshine led to an increase by 0.68 (95% CI 0.47% to 0.88%) in the monthly number of cases (Table 2).

**Table 2:** Negative binomial regression model of meteorological factors associated with risk of malaria inciedence

	**B**	**STD**	**P**	**(e**^**β**^ **− 1) = RR (%)**	**95% CI for RR (%)**	
					**Lower bound**	**Upper bound**
(A)						
Intercept	−350.15	16.80	0.02	-	-	-
Average atmospheric pressure	−0.01	0.01	0.60	−0.76	−3.60	2.15
Average relative humidity	0.02	0.01	0.05	2.42	−0.04	4.94
Average wind velocity	0.14	0.10	0.17	14.48	−5.62	38.86
Aggregate rainfall	0.00	0.00	0.93	0.00	−0.08	0.07
Aggregate Sunshine	0.01	0.00	0.00	0.52	0.24	0.80
year	0.02	0.03	0.54	2.11	−4.48	9.16
(B)						
Intercept	−560.76	21.02	0.01	-	-	-
Average temperature	0.01	0.06	0.03	0.80	0.40	3.77
Average relative humidity	0.02	0.01	0.04	2.31	0.15	4.53
Average wind velocity	0.17	0.10	0.11	18.03	−3.72	44.69
Aggregate rainfall	0.00	0.00	0.84	−0.01	−0.08	0.07
Aggregate Sunshine	0.00	0.00	0.00	0.48	0.19	0.77
year	0.03	0.04	0.44	2.81	−4.12	10.24
(C)						
Intercept	−11.69	0.52	0.00	-	-	-
Average temperature	0.01	0.01	0.03	0.90	0.60	1.10
Average relative humidity	0.04	0.01	0.00	3.99	2.53	5.48
Aggregate sunshine	0.01	0.00	0.00	0.68	0.47	0.88

*Note*.*Negative binomial regression model for monthly malaria incidence without average temperature (A) and without atmospheric pressure (B). Final models (C).

*RR, relative risk; CI, confidence Interval.

## Discussion and comment

Weather factors such temperature, humidity, et al. have been proved to have significant influence on occurrence and transmission of some infectious diseases. For example, in Switzerland, higher water vapor pressure and heat were found associated with a higher risk of community-acquired Legionnaires’ disease [10]; in Botswana, the elevation of annual minimum temperature was considered as the critical factor for continuous ascent in the number of diarrheal disease reported during the period of 1974–2003 [11]. The result of current study showed that temperature was positively associated with malaria incidence in Guangzhou area. This finding is in general agreement with other studies [12] in which temperature is considered to be a precipitating factor for distribution of Anopheles, the transmission vector of malaria. We found relative humidity was positively associated with malaria incidence of the same month. Similar observation was also reported in Cameroon where more Anopheles mosquitoes especially Anopheles gambiae were collected during the rainy season compared to the dry season [13,14]. Inconsistent with the study from Korea [13], we found duration of sunshine was positively associated with malaria incidence. However, this finding accords with Diego Ayala’s findings which showed malaria vectors occurrences were positively correlated to increasing sunlight exposure [15]. Further studies, which take these variables into account, will need to be undertaken.

Taken together, we have reported that weather factors had significant influence on occurrence and transmission of malaria in Guangzhou, southern China. A rise in temperature, relative humidity and duration of sunshine may increase the risk of malaria infection. Our finding provides preliminary but fundamental information that may be useful to develop a simple, precise, and low cost functional malaria early warning system.

## Competing interests

The authors declare no financial, academic or intellectual competing interests.

## Authors’ contributions

Conceived and designed the study: Zhicong Yang, Ming Wang. Analyzed the data: Tiegang Li. Contributed materials/analysis tools: Zhicong Yang, Ming Wang. Wrote the paper: Tiegang Li, Zhicong Yang, Ming Wang. All authors contributed to and approved the final version of the manuscript.

## Authors’ information

The authors are all epidemiologists in Guangzhou center for disease control and prevention (GZCDC). The authors regularly conduct the surveillance on infectious disease, filed investigation on outbreaks, emergent management on public health crisis, and research on risk factors and transmission of diseases.
